# A tale of two tumours: Comparison of the immune escape strategies of contagious cancers^☆^

**DOI:** 10.1016/j.molimm.2012.10.017

**Published:** 2013-09

**Authors:** Hannah V. Siddle, Jim Kaufman

**Affiliations:** a Department of Pathology, University of Cambridge, Tennis Court Road, Cambridge CB2 1QP, UK; b Department of Veterinary Medicine, University of Cambridge, Madingley Road, Cambridge CB3 0ES, UK

**Keywords:** Contagious cancer, Immune evasion, MHC, Tasmanian devil, Devil facial tumour disease, Canine transmissible venereal tumour, Cancer

## Abstract

The adaptive immune system should prevent cancer cells passing from one individual to another, in much the same way that it protects against pathogens. However, in rare cases cancer cells do not die within a single individual, but successfully pass between individuals, escaping the adaptive immune response and becoming a contagious cancer. There are two naturally occurring contagious cancers, Devil Facial Tumour Disease (DFTD), found in Tasmanian devils, and Canine Transmissible Venereal Tumour (CTVT), found in dogs. Despite sharing an ability to pass as allografts, these cancers have a very different impact on their hosts. While DFTD causes 100% mortality among infected devils and has had a devastating impact on the devil population, CTVT co-exists with its host in a manner that does not usually cause death of the dog. Although immune evasion strategies for CTVT have been defined, why DFTD is not rejected as an allograft is not understood. We have made progress in revealing mechanisms of immune evasion for DFTD both *in vitro* and *in vivo*, and here we compare how DFTD and CTVT interact with their respective hosts and avoid rejection. Our findings highlight factors that may be important for the evolution of contagious cancers and cancer more generally. Perhaps most importantly, this work has opened up important areas for future research, including the effect of epigenetic factors on immune escape mechanisms and the basis of a vaccine strategy that may protect Tasmanian devils against DFTD.

## Highlights

► There are two naturally occurring contagious cancers, Devil Facial Tumour Disease (DFTD) and Canine Transmissible Venereal Tumour (CTVT). ► Both contagious cancers share mechanisms of immune evasion. ► These mechanisms of immune evasion provide insights into how contagious cancers can emerge.

## 1 Introduction

In the late 1990s Tasmanian devils began to suffer from a disturbing disease that causes large, disfiguring tumours around the face and neck. This disease was defined as Devil Facial Tumour Disease (DFTD), and in 2006 Pearse and Swift proposed that these tumours all derived from a single neoplastic clone that was spreading as an allograft through the devil population (Pearse and Swift, 2006). This finding focused attention on the plight of a unique and vulnerable species, as well as the ability of cancers to escape the confines of a single individual. Comparisons were immediately drawn to the only other naturally occurring contagious cancer, Canine Transmissible Venereal Tumour (CTVT), a sexually-transmitted disease in dogs. Although these cancers are both contagious allografts, they are very different in their life history and biology. CTVT is a tumour that emerged thousands of years ago, whereas DFTD emerged relatively recently as it was first observed in 1996. Perhaps the most important difference is the contrasting relationship they have with their host. CTVT does not usually kill host dogs due to an evenly matched battle between tumour growth and the host immune response (Hsiao et al., 2008). In contrast, DFTD grows rapidly, invariably resulting in death of the infected devil from metabolic starvation, secondary infection, or organ failure following metastasis (Woods et al., 2007). Indeed, DFTD has had a devastating impact on the devils in Tasmania, with some populations declining as much as 90% and causing concern for the survival of the species in the wild (McCallum et al., 2007).

The contrasting impact that CTVT and DFTD have on their respective hosts is likely linked to how these cancers interact with the host immune system. However, while some aspects of the interaction between CTVT and the dog immune system are well characterised, very little is understood about how DFTD interacts with the devil immune system. This is due to its comparatively recent emergence, the paucity of immunological tools and reagents for Tasmanian devils, and difficulties associated with studying a wild species. We have recently made significant progress in defining immune escape mechanisms utilised by DFTD and can begin to understand how this tumour so successfully evades the devil immune response. These immune escape strategies reveal potentially important similarities to CTVT, enabling us to begin to understand how these tumours emerged, how they became so successful and perhaps where they are headed in the future.

## 2 Current status

The Tasmanian devil gained its common name from European settlers terrified by its nighttime vocalisations and misplaced reputation as an aggressive hunter. In fact, the Tasmanian devil is a scavenger and a solitary animal that interacts socially only when feeding and mating (McCallum et al., 2007). Tasmanian devils do regularly bite each other around the face and neck during feeding and mating interactions, but these fights are usually to establish hierarchy and do not usually result in mortal wounds. Prior to the emergence of DFTD, the Tasmanian devil was not considered threatened. The population had largely recovered from persecution inflicted by European settlers after their arrival in the 19th century and, as the top predator in the Tasmanian ecosystem (after the extinction of the Thylacine) and being well adapted to its environment, the Tasmanian devil population was stable (McCallum et al., 2007). However, in the late 1990s, DFTD emerged in a single devil in the northeast corner of Tasmania and these tumour cells gained the ability to move between individuals. It is thought that DFTD cells are physically passed between devils when they bite each other around the face and neck (McCallum et al., 2007). The tumour has since spread westward across Tasmania, with only the northwest corner of the island remaining disease free.

Perhaps unsurprisingly, very little was known about the devil immune system before the outbreak of DFTD, with only a limited number of immunological reagents and tools available. The first studies of the devil immune system found that these animals have a competent cellular and humoural immune response and should be able to respond to allograft cells such as DFTD (Woods et al., 2007). In order to understand why DFTD does not elicit a successful immune response from host devils attention turned to major histocompatibility complex (MHC) class I and class II molecules, due to the important role that these molecules play in graft rejection and the detection of malignant cells. MHC class I molecules are found on the surface of nearly all cells and present self and foreign peptides to CD8^+^ T cells. MHC molecules are generally highly polymorphic allowing a population to respond to many different pathogens and preventing graft transmission between unrelated individuals. Indeed, it has been hypothesised that the adaptive immune system emerged to protect multicellular organisms against parasitic cell lines, in much the same way as it protects against pathogens (Buss, 1982).

Although the Tasmanian devil population recovered after the arrival of European settlers, Tasmanian devils have reduced genetic diversity at neutral markers (reviewed in Jones et al., 2007). This reduced genetic diversity extends to the MHC class I and class II loci in devils, which have fewer alleles compared to other marsupial species (reviewed in Belov, 2011). The only clear population structuring across Tasmania exists between devils in the northwest of the island compared to the east, which is apparent at microsatellite and MHC loci. The reduction in genetic diversity at MHC loci led to the hypothesis that devils recognise DFTD cells as self due to similar MHC molecules between the host devil and DFTD cells. However, as DFTD has spread from east Tasmania towards the northwest, it has become apparent that DFTD successfully crosses histocompatibility barriers that would normally prevent allograft acceptance. In addition, allograft experiments between MHC mismatched and matched devils demonstrate their ability to reject allografts (Kreiss et al., 2011). As such, after intense focus on MHC genetics, it is apparent that a lack of genetic diversity at MHC loci cannot explain the transmission of DFTD.

We recently set out to determine the MHC molecules present on the surface of DFTD cells. To do this we needed to develop antibodies specific for devil MHC class I and β_2_-microglobulin (β_2_m) molecules. Using these antibodies we found that DFTD cells have few or no cell surface MHC class I molecules both on DFTD cells kept in culture and in primary tumour biopsies from wild devils. Loss of surface MHC class I is due to a down-regulation of β_2_m, TAP1 and TAP2 genes by DFTD cells. Interestingly, we can increase transcription of these genes *in vitro* by treating the cells with a histone deacetylase inhibitor, which suggests a role for epigenetic changes in the down-regulation of these genes. TAP1 and TAP2 form a heterodimer to pump peptides for the class I molecule into the endoplasmic reticulum (ER) and β_2_m stabilises the class I/peptide complex. We have shown that despite the presence of MHC class I heavy chain transcripts in the DFTD cells, there is very little MHC class I protein within the cells and on the cell surface. Thus, it is most likely that without β_2_m or peptide, the class I heavy chain is retained in the ER and degraded. These findings provide the first evidence that DFTD is actively evading the host immune system and indicates that MHC polymorphism does not have an affect on the spread of the tumour.

Compared to DFTD, CTVT is a very old contagious cancer that emerged thousands of years ago in wolves or one of the ancient breeds of dog, making it the oldest continuously passaged cell line in the world (Murgia et al., 2006; Rebbeck et al., 2009). Transmission of CTVT occurs during coitus, and tumours appear within two months after transmission (although tumours can appear faster in laboratory models). The initial growth stage of the tumours is termed the progressive phase (P), during which the immune system fails to control tumour growth. During the P phase, most CTVT cells lack expression of class I and class II molecules (MHC class I is found on only 3% of CTVT cells) and lymphocytes fail to infiltrate the tumour (Hsiao et al., 2008). This period of tumour growth does not continue indefinitely and after three to nine months the tumour either stabilises or begins to regress. Regression is characterised by significant increase in MHC class I and class II expression on the surface of the CTVT cells (MHC expression on 31% of cells), an infiltration of lymphocytes into the tumour mass and an increase in interferon-gamma (IFN-γ) production (Hsiao et al., 2008). Outside the laboratory setting, CTVT tumours often enter a stationary phase in which the tumour does not grow or regress. This homeostasis between tumour growth and the host immune system can last from months to years, providing ample time for the tumour to be passed to other dogs.

In common with CTVT, we have recently found that MHC expression can be restored to the surface of DFTD cells by treating these cells *in vitro* with IFN-γ, confirming that MHC loss in these cells is not due to structural mutations. We have also found evidence that MHC expression can occur on DFTD cells *in vivo*, where lymphocytes are clustered next to DFTD cells. However, this only seems to occur in rare cases and unlike CTVT, it does not impact the immune response to the tumour. This means that in contrast to CTVT, DFTD appears to be driving its host, and therefore itself, to extinction.

A shared characteristic of DFTD and CTVT is the lack of MHC class I and class II surface expression, which most likely represents one way in which these tumours avoid host immune defenses. However, the way in which these tumours down-regulate the expression of MHC molecules has common elements that may be important for the emergence of contagious cancers. Many tumours lose expression of MHC molecules permanently due to structural mutations in MHC genes. Both CTVT and DFTD have lost MHC expression, but this is due to regulatory mechanisms, not structural mutations. However, for CTVT, MHC expression is lost and then restored, resulting in a balance between the immune response and tumour growth that is key to its continual survival as it allows the host immune system to control the cancer. This is not the case for DFTD, but the ability of DFTD cells to regulate their MHC molecules gives this tumour the potential to up-regulate MHC expression over time. This may effect the evolution of the cancer and allow it to evolve into less aggressive subtypes. For example, a slower growing subtype of DFTD may allow the devil immune system more time to target tumour cells, causing a release of IFN-γ, up-regulation of MHC expression by DFTD cells and subsequent control of the tumour by the devil immune system.

## 3 Future directions

The loss of MHC expression in single organism tumours is usually associated with immune escape and metastasis, and can be due to either structural mutations or regulatory mechanisms such as epigenetic changes affecting gene transcription. Structural mutations leading to loss of MHC expression are usually easily identified, and in most cases are irreversible. In contrast, the cause of coordinated down-regulation of multiple genes in the antigen processing and presentation pathway can be more difficult to define. DFTD and CTVT clearly lose MHC expression *via* regulatory mechanisms as both tumours can up-regulate MHC expression *via* the IFN-γ pathway. However, in both tumours the exact mechanisms of gene suppression are not yet clear.

In recent years the importance of epigenetic changes in the transformation to malignancy has been increasingly appreciated (Setiadi et al., 2007). Our work suggests that epigenetic mechanisms are affecting MHC expression by DFTD cells. These epigenetic changes most likely involve the modification of histones or changes in the binding of transcription factors. Of course, these two factors may be related to one another, as histone acetylation is often required for binding of transcription factors to promoters. CTVT cells down-regulate MHC class I heavy chain transcripts, but the expression of β_2_m and the TAP genes has not been examined at the molecular level and again the mechanisms suppressing MHC class I expression are largely unknown. More work is needed to determine if and how epigenetic mechanisms, including chromatin modifications, are affecting MHC expression in both DFTD and CTVT.

The loss of MHC molecules from the surface of CTVT and DFTD cells should cause a response from NK cells as the inhibitory signal is lost from these cells. It is thought that NK cells do not target CTVT cells during the P phase of growth due to the release of TGFβ by CTVT cells that suppresses the response of NK cells. Why DFTD cells are not subject to lysis by NK cells is not yet understood, but the balance of activating and inhibitory NK ligands could be controlled by regulatory mechanisms, as we have found for MHC molecules.

Our ability to restore MHC molecules to the surface of DFTD cells using IFN-γ provides an opportunity develop a whole cell vaccine to DFTD. The MHC molecules and peptides presented by DFTD cells will be foreign to most if not all host devils and should trigger an immune response. Host devils will then be activated against these foreign antigens even if found at only low levels on DFTD cells, as well as intracellular antigens released by DFTD cells during tumour transmission and growth. The induction of any immune response to DFTD should trigger the release of IFN-γ, which in turn should up-regulate MHC expression on DFTD cells. We are hopeful that a whole cell vaccine will tip the balance between the growth of DFTD and the immune response in favour of the devil.

Principles of selection and evolution apply to tumours as a heterogeneous group of cells that are competing against each other for space and resources, as well as against the host immune system. When tumour cells metastasise this event is usually associated with immune escape. In a sense, the movement of DFTD cells between individuals is a metastatic event and therefore new selective pressures should be applied to the tumour cells with each individual it is passed through. DFTD has only emerged recently and already there is evidence of selection occurring, leading to the emergence of a variety of tumour subtypes (Murchison et al., 2012). There is as yet no information on the functional differences (if there are any) between these DFTD subtypes. However, further investigation may reveal that the immune response to DFTD varies depending on the subtype. It is also possible that one of these different subtypes will eventually modulate its MHC expression, leading to a less aggressive cancer.

Given that tumours arise by escaping the immune response, often facilitated by the loss of MHC molecules from the surface of a tumour cell, it is surprising that contagious cancers arise so rarely. There are examples of tumours passing from one individual to another in humans, usually as a result of a maternal tumour passing to a foetus or transplantation, but these tumours spread no further. Thus, there must be pre-requisites in place for contagious cancers to emerge. These pre-requisites may include behaviour, genetic features of a population and/or the cell type of origin for the cancer. DFTD is thought to have arisen from a Schwann cell (Murchison et al., 2010) and CTVT is thought to have arisen from a macrophage cell (Murgia et al., 2006). Aspects of these cell types may predispose them towards immune escape phenotypes, and this is an area of research that requires further attention.

The recent progress we have made in understanding how DFTD escapes the host immune response allows comparisons to be made between CTVT and DFTD as to how they interact with the host immune system and how each tumour progresses after transmission. While DFTD and CTVT modulate MHC expression in a similar manner, disease progression in the two tumours is very different, suggesting that we do not have the full story of how DFTD evades the immune response. Further investigation of these tumours promises to reveal how contagious cancers can emerge, how they escape the immune response and how this impacts the complex relationship they have with their host species.

## References

[bib0005] Belov K. (2011). The role of the major histocompatibility complex in the spread of contagious cancers. Mammalian Genome.

[bib0010] Buss L.W. (1982). Somatic cell parasitism and the evolution of somatic tissue compatibility. Proceedings of the National Academy of Sciences of the United States of America.

[bib0015] Hsiao Y.W., Liao K.W., Chung T.F., Liu C.H., Hsu C.D., Chu R.M. (2008). Interactions of host IL-6 and IFN-gamma and cancer-derived TGF-beta1 on MHC molecule expression during tumor spontaneous regression. Cancer Immunology Immunotherapy.

[bib0020] Jones M.E., Jarman P.J., Lees C.M., Hesterman H., Hamede R.K., Mooney N.J., Mann D., Pukk C.E., Bergfeld J., McCallum H. (2007). Conservation management of Tasmanian devils in the context of an emerging, extinction-threatening disease: devil facial tumor disease. EcoHealth.

[bib0025] Kreiss A., Cheng Y., Kimble F., Wells B., Donovan S., Belov K., Woods G.M. (2011). Allorecognition in the Tasmanian devil (*Sarcophilus harrisii*), an endangered marsupial species with limited genetic diversity. PLoS ONE.

[bib0030] McCallum H., Tompkins D.M., Jones M.E.S.L., Marvanek S., Lazenby B., Hocking G., Wiersma J., Hawkins C.E. (2007). Distribution and impacts of Tasmanian devil facial tumor disease. EcoHealth.

[bib0035] Murchison E.P., Schulz-Trieglaff O.B., Ning Z., Alexandrov L.B., Bauer M.J., Fu B., Hims M., Ding Z., Ivakhno S., Stewart C., Ng B.L., Wong W., Aken B., White S., Alsop A., Becq J., Bignell G.R., Cheetham R.K., Cheng W., Connor T.R., Cox A.J., Feng Z.P., Gu Y., Grocock R.J., Harris S.R., Khrebtukova I., Kingsbury Z., Kowarsky M., Kreiss A., Luo S., Marshall J., McBride D.J., Murray L., Pearse A.M., Raine K., Rasolonjatovo I., Shaw R., Tedder P., Tregidgo C., Vilella A.J., Wedge D.C., Woods G.M., Gormley N., Humphray S., Schroth G., Smith G., Hall K., Searle S.M., Carter N.P., Papenfuss A.T., Futreal P.A., Campbell P.J., Yang F., Bentley D.R., Evers D.J., Stratton M.R. (2012). Genome sequencing and analysis of the Tasmanian devil and its transmissible cancer. Cell.

[bib0040] Murchison E.P., Tovar C., Hsu A., Bender H.S., Kheradpour P., Rebbeck C.A., Obendorf D., Conlan C., Bahlo M., Blizzard C.A., Pyecroft S., Kreiss A., Kellis M., Stark A., Harkins T.T., Marshall Graves J.A., Woods G.M., Hannon G.J., Papenfuss A.T. (2010). The Tasmanian devil transcriptome reveals Schwann cell origins of a clonally transmissible cancer. Science.

[bib0045] Murgia C., Pritchard J.K., Kim S.Y., Fassati A., Weiss R.A. (2006). Clonal origin and evolution of a transmissible cancer. Cell.

[bib0050] Pearse A.M., Swift K. (2006). Allograft theory: transmission of devil facial-tumour disease. Nature.

[bib0055] Rebbeck C.A., Thomas R., Breen M., Leroi A.M., Burt A. (2009). Origins and evolution of a transmissible cancer. Evolution.

[bib0060] Setiadi A.F., David M.D., Seipp R.P., Hartikainen J.A., Gopaul R., Jefferies W.A. (2007). Epigenetic control of the immune escape mechanisms in malignant carcinomas. Molecular and Cellular Biology.

[bib0065] Woods G.M., Kriess A., Belov K., Siddle H.V., Obendorf D.L., Muller H.K. (2007). The Immune response of the Tasmanian devil (*Sarcophilus harrisii*) and devil facial tumour disease. Ecohealth.

